# Misunderstanding of race as biology has deep negative biological and social consequences

**DOI:** 10.1113/EP091491

**Published:** 2024-05-03

**Authors:** Heidi L. Lujan, Stephen E. DiCarlo

**Affiliations:** ^1^ Department of Physiology, College of Osteopathic Medicine Michigan State University East Lansing Michigan USA

The belief of race as a biological concept with human populations having a distinct biological basis is unconsciously embedded in many individuals’ thinking (Lee, et al., 2021). These individuals think population differences in health and intelligence are the result of immutable, biologically based differences between ‘racial’ groups, despite overwhelming evidence that racial groups are not genetically discrete, reliably measured or scientifically meaningful (Smedley & Smedley, 2005). Because racial groups are not genetically discrete, when the term ‘race’ is discussed, we are referring to the self‐identified races (socio‐cultural constructs)—and not race as a biological entity.

Although racial groups are not genetically separate, a substantial number of American medical students, residents, and physicians hold false beliefs about biological differences between races. A survey of 222 white American medical students and residents, using true and false statements about the biological differences between Blacks and Whites, reported that 40% of first‐ and second‐year medical students believed the statement that ‘The skin of a black person is thicker than a white person’ (Hoffman et al., 2016). About 25% of residents also thought that black skin was thicker. In addition, because of the false belief that Blacks are more tolerant of pain, Whites (74%) were more likely to be prescribed analgesics than Blacks (50%) in the emergency department of an Atlanta area hospital (Josefson, 2000; Lee, et al., 2018). The belief that white individuals and black individuals differ in their response to the same medical treatment is also widespread in the medical profession (Green et al., 2007; Jha et al., 2005; Schulman et al., 1999; van Ryn, 2002; van Ryn et al., 2006). In 2005, the Food and Drug Administration approved the heart failure drug, BiDil, (isosorbide dinitrate/hydralazine—a fixed dose combination drug) for use specifically by African Americans, even though there was no evidence that it had any racially disparate effects (Kahn, 2013). This led investigators to conclude ‘The scientific research leading to BiDil's approval tested the drug only in African American populations, apparently for commercial reasons, so the drug's efficacy in other populations is unknown’ (Brody & Hunt, 2006).

Similarly, the results of a systematic review of 15 studies documented that implicit racial bias was significantly related to patient–provider interactions, treatment decisions, treatment adherence and patient health outcomes (Hall et al., 2015). Thus, a substantial number of physicians, medical students and residents hold false beliefs about biological differences between races and these beliefs predict racial bias and racial disparities in pain perception and treatment recommendation accuracy (Ayanian et al., 1999; Green et al., 2007; Hoffman et al., 2016; Jha et al., 2005).

The misunderstanding of race as biology is not limited to the United States as this has been documented globally (Outram et al., 2018). One example includes university students majoring in biology and medicine (Lee et al., 2021; Lee, et al., 2018). Over 40% of Korean college students majoring in General Science Education believed the concept of race from a biological perspective (Lee et al., 2021). Similarly, nearly 40% of Korean medical students held a biological or genetic concept of race (Lee, et al., 2018). Some Korean medical students defended their belief that race is a biological reality by citing various statistics showing different rates of disease among different groups (Lee, et al., 2018).

Misunderstanding of race as biology may be due, in part, to the observation that race is often operationalized as a biological concept in some medical school's teaching, and some existing educational materials may reinforce institutional racism within medical education (Johnson et al., 2017; Tsai et al., 2016). An analysis of a question bank for Step 1 of the United States Medical Licensing Examination revealed questions that contained racially biased information (Ripp & Braun, 2017). Similarly, a review of lecture slides at a major medical school demonstrated that race was almost always presented as a biological risk factor and framed racial health disparities as inherent biological differences (Tsai et al., 2016). A review of case studies from a medical school in the United States revealed a strong racial bias when describing the patient and pathology (Johnson et al., 2017). Thus, reports suggest an implicit bias, discrimination and racism embedded in our medical education curricula (Ansell & McDonald, 2015) which may contribute to the misunderstanding of race as biology. It must also be noted that a substantial number of faculty members surveyed from 12 colleges and universities across the United States reported that racial bias may be affecting student's mental health (Lipson, 2023). Overall, 25% of faculty believe their institution is ‘hostile’ or ‘somewhat hostile’ toward students of colour. A total of 58% of Hispanic or Latinx faculty, 39% of Black or African American faculty, and 24% of Asian or Asian American faculty believe their institution is ‘hostile’ or ‘somewhat hostile’ toward students of colour.

However, biological races do not exist among humans (Smedley & Smedley, 2005). Although it is well accepted that ancestral alleles [alleles from people you directly descend from (parents, grandparents)] can affect disease rates and medication efficacy (Ashley‐Koch et al., 2000), these alleles do not align with racial groupings. Specifically, admixture and migration have produced broad variation so that race categories cannot substitute for genetic ancestry (Bamshad et al., 2004; Bolnick, 2008; Fujimura & Rajagopalan, 2011; Fujimura et al., 2010; Graves & Rose, 2006; Hunt & Megyesi, 2008; Kalinowski, 2011; Tang et al., 2006) and there are no sharp boundaries dividing the human species, as gene frequency differences between populations found in one polymorphism are not paralleled in others (Barbujani, 2005; Madrigal & Barbujani, 2007; Serre & Paabo, 2004; Tishkoff & Kidd, 2004). Accordingly, studies document that genetic differences are far higher within than between racial groups (Lewontin, 1972). Thus ‘race’ is not a biological reality but a myth (Smedley & Smedley, 2005). It is not possible to categorize an entire racial group as being genetically or physiologically distinct from one another because of the vast genetic heterogeneity of our populations.

Furthermore, there is no accepted scientific method for classifying people by race (Kurtz et al., 2021). Studies typically rely on self‐identification of race (Lujan & DiCarlo, 2021). However, the distinctions and nuances of racial identity, geographic origin, and history in the identification of races are misunderstood. ‘African American’ refers specifically to people with American nationality and African geographic ancestral origin (Tsai et al., 2016). ‘Black’ refers to people with black skin phenotype, which includes all nationalities, as well as geographic origins (Tsai et al., 2016). These labels should not be used interchangeably because doing so confuses ancestry with social geography. It is also important to note that ethnicity and culture are related phenomena but bear no intrinsic connection to human biological variations or race (Smedley & Smedley, 2005). Ethnic groups and ethnicity are not fixed, bounded entities; they are open, flexible, and subject to change, and they are usually self‐defined (Smedley & Smedley, 2005).

The presentation of race as inherent biological differences is polarizing because it strengthens existing racial biases (Condit et al., 2004) and creates confusion and misunderstandings. The misunderstanding of race as biology provides a rationale for dividing people, and treating black individuals as a distinctly different genetic group (Kaufman & Hall, 2003; Lujan & DiCarlo, 2018a) and it supports our tribal bias (Johnson et al., 2017; Lujan & DiCarlo, 2018b) for Black inferiority that is innately pathological. This is not new. Historically, populations who have dominated others have regarded themselves as natural superiors. Explicitly, all populations in positions of advantage culturally and economically have looked upon others as their inferiors and have resented any claims on the part of the reputed inferiors to social or biological equality (Reinhardt, 1927).

The polarizing myth of genetic inferiority of people of colour has a deep negative impact on students. When students read or hear this myth often enough, sooner or later they begin to believe what they hear or read, whether it is true or not. This is an example of the Barnum or Forer effect or ‘acceptance phenomenon’, which describes the general tendency of humans ‘to accept almost any bogus personality feedback’ (Tobacyk et al., 1988).

Consider that minority students have a higher rate of medical school withdrawal and dismissal, as well as lower pass rates on the US Medical Licensing Examination Step 1 test (Andriole & Jeffe, 2010; Carr & Woodson, 2008). Furthermore, underrepresented minority students have reported less supportive social and less positive learning environments (Orom et al., 2013). Minority students have also expressed that perceptions of their race have negatively impacted their medical school training at higher rates than non‐minority students (Dyrbye et al., 2007). Thus, correcting the misunderstanding of race as biology and refusing to treat black individuals as a distinctly separate group may help to provide a welcoming and safe environment that results in retaining more minority students, and, by extension, improving the health care of minority populations.

Specifically, evidence of racial disparities in the access to health care, the quality of care received, and health outcomes are well documented; people of colour receive less and lower quality healthcare than Whites (Ansell & McDonald, 2015; Edwards, 2003; Spector, 2004; Sullivan, 2004). Data from 47,291 adults aged 18 years or older who participated in the 2021 National Survey on Drug Use and Health, documented that Black adults were substantially less likely to receive medication for opioid use disorder (Jones et al., 2023). Thus, race plays an important role in determining how individuals are treated. However, healthcare is improved when it is provided by someone of the same ethnic and cultural background (Brown et al., 2005; Cooper‐Patrick et al., 1999; Spensler et al., 2003). Accordingly, retaining more minority students may improve the health care of minority populations.

The use of race in genomics and genetics research as a surrogate for describing human genetic differences is confusing, mistaken and damaging. Similarly, the tendency for race to be classified as a biological covariate when investigating the incidence and prevalence of diseases among different groups is a serious concern. Race is complicated by social class, sex, geographical context and socio‐economic demographics. However, importantly, being considered part of a race has biological effects. Specifically, being considered part of a race can affect exposure to environmental toxins, rates of incarceration, and living in poor neighbourhoods with limited access to healthy and affordable food. Even experiencing the stress of racial discrimination can have biological effects. Thus, race is a social category that can have biological consequences (Smedley & Smedley, 2005). Accordingly, we must provide social contextualization when discussing the incidence and prevalence of diseases among different groups. For example, although we should study and document group disparities in asthma among children, these data must also include socioeconomic considerations because teaching these disparities without teaching context may suggest inherent biological differences (Tsai et al., 2016) and by extension, influence patient–provider interactions, treatment decisions, treatment adherence and patient health outcomes.

In conclusion, a misunderstanding of race as biology continues to play a role in medical education and research. This misunderstanding has deep negative biological and social consequences. Accordingly, we should stop dividing people, reject the premise of genetic determinism, and not think of racial groups as inherently distinct in terms of biology or physiology. We should also purge educational materials that polarize and reinforce institutional racism within medical education (Lim et al., 2021). Overcoming our bias and refusing to treat black individuals as a genetically homogeneous group is critical for our students and the nation's health.

## AUTHOR CONTRIBUTIONS

Heidi L. Lujan: conception or design of the work; drafting of the work or revising it critically for important intellectual content. Stephen E. DiCarlo: conception or design of the work; drafting of the work or revising it critically for important intellectual content. All authors have read and approved the final version of this manuscript and agree to be accountable for all aspects of the work in ensuring that questions related to the accuracy or integrity of any part of the work are appropriately investigated and resolved. All persons designated as authors qualify for authorship, and all those who qualify for authorship are listed.

## CONFLICT OF INTEREST

None of the authors have any conflicts of interest.

## FUNDING INFORMATION

No funding was received.
